# A new species of *Eumerus* (Diptera, Syrphidae) from the Kingdom of Bhutan, the easternmost representative of the *bactrianus* subgroup

**DOI:** 10.3897/zookeys.906.48501

**Published:** 2020-01-27

**Authors:** John Smit, Theo Zeegers, Phurpa Dorji

**Affiliations:** 1 Naturalis Biodiversity Center / European Invertebrate Survey – the Netherlands, PO Box 9517, 2300 RA Leiden, the Netherlands Naturalis Biodiversity Center Leiden Netherlands; 2 Project Officer, Gross National Happiness Commission, Thimphu, Bhutan Gross National Happiness Commission Thimphu Bhutan; 3 The Royal Society for Protection of Nature, Thimphu, Bhutan The Royal Society for Protection of Nature Thimphu Bhutan

**Keywords:** Central Palaearctic, Eastern Palaearctic, flower flies, hover flies, Himalaya, identification key

## Abstract

A new species of *Eumerus*, *Eumerus druk* Smit **sp. nov.**, is described from Bhutan. This species belongs to the *bactrianus* subgroup of the *strigatus* species group. Seven species are currently known within this subgroup: four European, one of which is also found in the Near East, and three more only known from Tajikistan. The new species extends this disjunct distribution to the east by at least 2,000 km, stretching far beyond the reported Turano-Mediterranean region and into the Himalayas. A diagnosis and a key to all Central and Eastern Palaearctic species of the *Eumerus bactrianus* subgroup are provided.

## Introduction

Hover flies are often large and attractively coloured insects that are frequently found on flowers and play a vital role in ecosystem services as pollinators (Biesmeijer et al. 2006; Inouye et al. 2015; Ssymank et al. 2008). In contrast, the majority of the species from the very speciose genus *Eumerus* Meigen, 1822 are inconspicuously dark coloured, sometimes with metallic bronze-green or even golden luster or bluish sheen, and they are more often than not found on the ground or on rocks. With the wings folded over the abdomen, the metallic luster is obscured when in rest. For the same reason, the bright-red abdomen of some species is less conspicuous when in rest (J. Smit pers. obs.). The genus is widespread in the Old World and Australia and introduced to the Americas (Johnson 1910; Davidson 1915; Smith 1928; Gerding et al. 1999; Marinoni and Morales 2007; Speight et al. 2013). There are over 300 valid species of *Eumerus* described (Evenhuis and Pape 2019) and taxonomical difficulties abound, mainly due to the large number of species as well as the lack of comprehensive keys. Fortunately, in recent times more and more species groups have been treated integrally, where morphology is often supplemented by molecular characters (Grković et al. 2015, 2017, 2019; Chroni et al. 2017, 2018). One such species cluster is the *bactrianus* subgroup of the *Eumerus strigatus* group as defined by Grković et al. (2019). This subgroup has four Western Palaearctic described species (Markov et al. 2016; Grković et al. 2019) and three species only known from the Hissor mountain range (‘Gissar’ in Russian) in Tajikistan (Stackelberg 1952). In their work, Grković et al. (2019) treated the Western Palaearctic representatives from this subgroup and redescribed *Eumerus bactrianus* Stackelberg, 1952, one of the species from Tajikistan. In this paper we describe a new Eastern Palaearctic species from Bhutan. The discovery of this new species from Bhutan, a small kingdom in the eastern Himalayas, stretches the known distribution of the *bactrianus* subgroup some 2,000 km to the east, well beyond the reported Turano-Mediterranean region by Grković et al. (2019). Diagnosis and an identification key to all four Central and Eastern Palaearctic species are presented.

This new species was collected during an expedition in spring 2018 as part of the Bhutan Biodiversity Project. This project is a cooperation between the National Biodiversity Center (Bhutan), Naturalis Biodiversity Center (Netherlands), and five other Bhutanese organizations aiming to generate knowledge on Bhutanese invertebrates. The main goal was to make a survey of several invertebrate groups and make this knowledge available through publications and field guides.

## Material and methods

Material from the following collections has been studied or is deposited therein, introducing the abbreviations: National Biodiversity Center, Thimphu, Bhutan (NBCB), Naturalis Biodiversity Center, Leiden, the Netherlands (NBC), and Zoological Institute in St Petersburg, Russia (ZIN). Male genitalia were removed and macerated in an aqueous 10% KOH solution at ambient temperatures for 12–24 hours and stored in glycerol. Photos of the terminalia were taken through a Bresser Biolux NV microscope with a MicrOculair and CamLabLite software, and subsequently stacked using Combine ZP 1.0 software. The remaining photos were made using an Olympus Tough TG-5 camera with built-in focus stacking software. The male holotype and the female paratype of *Eumerus druk* Smit sp. nov. had one leg removed for DNA barcoding (Hebert et al. 2003a, 2003b). DNA barcodes, sequences and collection data were uploaded to the Barcode of Life Database (BOLD: http://www.boldsystems.org). Specimens are linked through their specimen code to their respective entry on BOLD. Terminology of morphological characters follows Thompson (1999), with the exception of the terminology for the genitalia, which follows Doczkal (1996) and Hurkmans (1993). Abdominal tergites and sternites are abbreviated with a ‘t’ or ‘s’ respectively.

## Taxonomy

The *Eumerus strigatus* group was first defined by Speight et al. (2013) for a group of species closely related to *E. strigatus* (Fallén, 1817), i.e., *E. consimilis* Simic & Vujic, 1996, *E. funeralis* Meigen, 1822, *E. narcissi* Smith, 1928, *E. sogdianus* Stacklberg, 1952, and *E. strigatus*. Later Chroni et al. (2017) added *E. amoenus* Loew, 1848 based on molecular data and Grković et al. (2017) included another two species (i.e., *E. montanum* Grković, Radenković & Vuijć, 2017 and *E. pannonicus* Ricarte, Vujić & Radenković, 2016). Grković et al. (2017) provided a description and a diagnosis for the group, stating that it comprises relatively small, inconspicuous species with usually a bronze shine and without coloured markings on the tergites, simple sternites, and s4 in males differently shaped but always with a v-shaped notch at the posterior margin. The main diagnostic character is the shape of the male genitalia, particularly the epandrium with an elongated, posterior surstyle lobe of a species-specific shape.

The *Eumerus bactrianus* subgroup within the *strigatus* group was defined by Grković et al. (2019), and its members are very similar to the other species of the *strigatus* group but share the apomorphic character of the bifurcate posterior lobe of the surstylus in the male terminalia. Furthermore, the shape of s4 is more complex in the *bactrianus* subgroup than in the other species of the *strigatus* group. All species of the *bactrianus* subgroup are easily recognised; all have a unique shape of their antennae, s4, and the male terminalia. Females of all species of the *bactrianus* subgroup as well as the females of the *strigatus* group are extremely similar. The females of all species from the Central Palaearctic are known but have not been examined; therefore, the identification key presented here is only for the males.

The *bactrianus* subgroup is represented by four Western Palaeartic species (i.e., *E. banaticus* Nedeljković, Grković & Vujić, 2019, *E. bicornis* Grković, Vujić & Hayat, 2019, *E. bifurcatus* van Steenis & Hauser, 2019, and *E. pannonicus*), three Central Palaearctic species described from the Hissor Mountains in Tajikistan (Stackelberg 1952) (i.e., *E. bactrianus*, *E. turanicola* Stackelberg, 1952, and *E. turanicus* Stackelberg, 1952), and one Eastern Palaearctic species, *E. druk* Smit sp. nov. from Bhutan. Of the Western Palaearctic species, only *E. bicornis* is also found outside Europe, more precisely in Turkey in the Near East.

The Palaearctic Region can be divided into subregions. Semenov-Tian-Shanskij (1936) made a first division in four subregions based on the distribution of Coleoptera, combined with the geological history as well as the fossil fauna. This only appeared in Russian, but an English summary was published in Nature that same year (Anonymous 1936). Kozár (1995) modified it and now the current subdivision into three regions, Western, Central, and Eastern, is widely applied (Kodandaramaiah and Wahlberg 2009; Sanmartin et al. 2001; Simonsen et al. 2010).

### Eumerus bactrianus

Taxon classificationAnimaliaDipteraSyrphidae

Stackelberg, 1952

CE66AC74-CCED-527E-95C7-458AC9F624C3

#### Material examined.

Paratype Tajikistan • male; у. Копдара 1100 m, д. ВарзобаТадж., Гуссаковский [Kopdara 1100 m, d. VarzobaTadž., Gussakovskii]; 15 May [19]39 (ZIN).

#### Diagnosis.

Body golden- or bronze-green, often with purple tinge. Legs bronze-green, tip of femora and basal third of tibia as well as tarsomeres 1–4 brightly brownish yellow, apical tarsal segment dark. Metaleg with basotarsomere expanded and short, as longs as second and third segment combined. Basoflagellomere trapezoid (Fig. 2F). s4 rectangular, roughly wrinkled, posterolateral narrowly rounded corners with long pile, posteromedially with a deep and sharp notch. Male terminalia figured in Grković et al. (2019: fig. 7A–D), anterior sustyle lobe elongated, ventral margin of posterior surstyle lobe greatly produced.

### Eumerus druk

Taxon classificationAnimaliaDipteraSyrphidae

Smit sp. nov.

A8F9117E-238E-5B4C-A4BA-B729423A5FDA

http://zoobank.org/5BBC6AD2-F2BF-4726-A26E-6055791AFE79

Figs 1A–F , 2A–E, G

#### Type locality.

Bhutan, Thimphu.

#### Diagnosis.

Body golden-coppery, except t2 and t3 medially and t4 basomedially: shiny black amplified by short adpressed black pile. Basoflagellomere rectangular, with a rounded posterior corner. Male: abdomen t3 and t4 laterally with long, silvery, ventrally directed pile; s4 without an incision posteriomedially but medial part of sternite less sclerotized. Basotarsomere of metaleg simple, equal in length to the rest of the tarsomeres. Male terminalia: posterior surstyle lobe with a tuft of long pili just anterior to the bifurcation.

#### Description.

**Figure 1.**
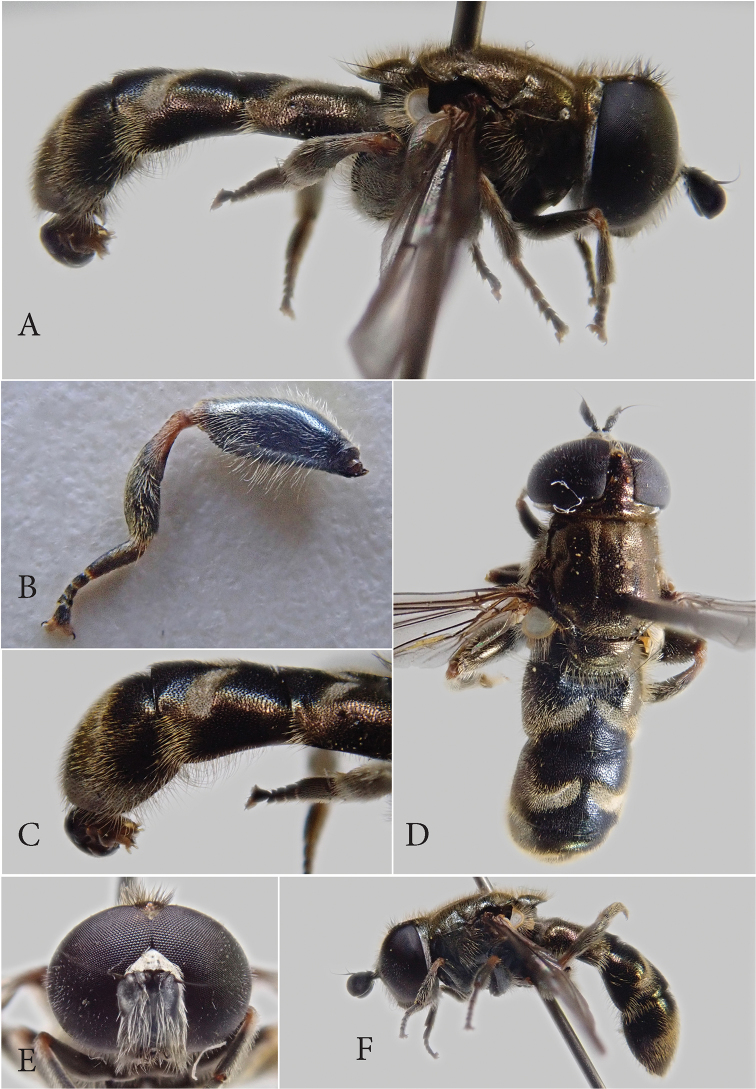
*Eumerus druk* Smit, sp. nov., male holotype **A** lateral view **B** metaleg, lateral view **C** abdomen, lateral view **D** dorsal view **E** head, frontal view. *Eumerus druk* Smit, sp. nov., female paratype **F** lateral view.

**Figure 2.**
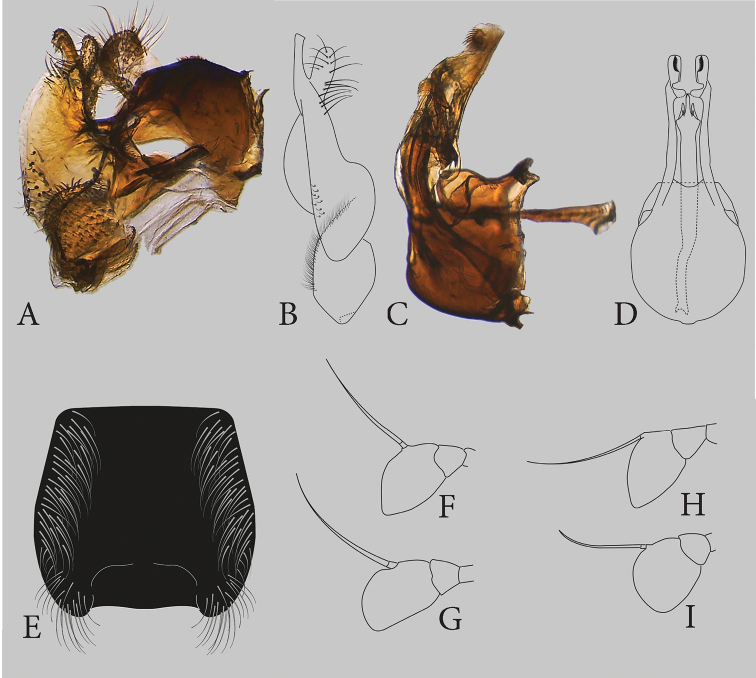
*Eumerus druk* Smit, sp. nov., male holotype **A** epandrium, lateral view **B** surstyle lobe, ventral view **C** hypandium, lateral view **D** hypandrium, ventral view **E** fourth sternum. *Eumerus bactrianus* Stackelberg, 1952, male **F** antenna, lateral view. *Eumerus druk* sp. nov. **G** antenna, lateral view. *Eumerus turanicola* Stackelberg, 1952, male **H** antenna, lateral view, after (Stackelberg 1952). *Eumerus turanicus* Stackelberg, 1952, male **I** antenna, lateral view, after (Stackelberg 1952).

**Male.** Length of body (excluding antennae) 7.5–8.5 mm, length of the wing 5.5–6.5 mm. ***Head*.** Eyes holoptic, eye contiguity 9–10 ommatidia long, ommatidia near eye contiguity conspicuously larger than those in the posterior part (Fig. 1E). Eye margins ventrally slightly divergent. Eye covered with dense white pile; posterior eye margin bare. Face with dense, silvery-white pollinosity and white pile. Frons with golden-yellow pile, intermixed with black pile or even predominantly black pilose on the ocellar triangle. Ocellar triangle isosceles; distance between anterior ocellus and posterior ocelli compared to the distance between both posterior ocelli 1:0.55. Frons with a small pollinose macula anterior to anterior ocellus. Occiput with dense white pollinosity up to about 3/4 dorsally; dorsal part shiny black, with coppery luster. Antenna black; basoflagellomere rectangular (Fig. 2D), with a rounded posterior corner. Arista entirely black. Scape and pedicel black, with white pile; black pile dorsally; dorsal pile much shorter than ventral pile.

***Thorax*.** Entirely shiny black, with golden luster (Fig. 1D). Mesonotum with a pair of white pollinosity vittae covering 3/4 of scutal length. Mesonotum and scutellum covered with golden-yellow pile; clearly longer near the posterior margin of the mesonotum and scutellum. Notopleural suture absent. Scutum next to wing base with a row of strong black setae. Scutellum with a broad rim, somewhat granular. Anepisternum and anepimeron with the same golden luster; katepisternum pollinose, with a small shiny spot dorsally, posterior to tuft of long white pile, ventrally with a few long white pili. ***Legs*.** All black, except for the tibiae, which are red on the basal third. Tarsi black, claws bicoloured, red basally, and black apically. Metafemur moderately swollen, slightly curved, with two rows of black setae apicoventrally, 11 on anterior ridge and 11–13 on posterior ridge, long white pile dorsally, about half as long as the maximum width of the femur and even longer white pile ventrally, the longest ones slightly more than 3/4 the maximum width (Fig. 1B). Metatibia with a flange of adpressed setae on the basal half, ventrally, followed by a shallow notch, apicoposteriorly with a single row of long light pile, longer than the maximum width of the metatarsus. Basotarsomere of metaleg simple, equal in length to the rest of the tarsomeres. ***Wings*.** Hyaline, pterostigma light brown, entirely microtrichose.

***Abdomen*.** Entirely black, parallel sided, t2–4 with oblique maculae of white pollinosity, those on t3 and t4 longer and clearly lunulate (Fig. 1A). t2 and t3 shiny black medially, as well as t4 basomedially, laterally with golden-coppery luster (Fig. 1D). The black colour in the middle of the tergites is amplified by the short adpressed black pile, light on the pollinose maculae as well as on the lateral sides and the majority of the t4. Abdomen with conspicuous long, silvery, ventrally directed, white pile on the lateral sides of the t3 and t4 (Fig. 1C). s4 with long silvery-white pile laterally, distinctly shorter medially, posteromedially without incision, but medial part of sternite less sclerotized (Fig. 2E).

***Terminalia*** (Fig. 2A–D). Posterior lobe of sursylus bifurcate, with a tuft of long light pile just anterior to bifurcation.

#### Description of female.

Similar to male except for the normal sexual dimorphism (Fig. 1F). Length of body (excluding antennae) 7 mm, length of the wing 6 mm. ***Head*.** Frons with some pollinosity alongside the eye-margin, from the antennae up to the anterior ocellus. Ocellar triangle isosceles, distance between anterior ocellus and posterior ocelli compared to the distance between both posterior ocelli 1:0.88. ***Abdomen.*** t3 and t4 laterally with slightly longer, silvery and ventrally directed, pile.

#### Etymology.

The specific epithet ‘druk’ is Dzongkha (the Sino-Tibetan language spoken in Bhutan) for dragon and refers to the official name of the kingdom: *Druk yul* (country of the Dragon people, or the Land of the Thunder Dragon). It should be treated as a noun in apposition.

#### Distribution.

This species is only known from the type series collected at the Royal Botanical Garden in Thimphu, Bhutan, but it likely has a wider distribution in the Himalayas. This is the only Eastern Palaearctic species of the *bactrianus* subgroup of the *strigatus* species group.

#### Examined material.

Type material. ***Holotype*** Bhutan • male; Thimphu, Royal Botanical garden; 27.425N, 89.650E, 2400 m a.s.l.; 26 April 2018; J.T. Smit & Th. Zeegers leg.; RMNH.INS1092470.

***Paratypes*** Bhutan • 4 males; same collection data as for holotype • 1 female; same data as for holotype; RMNH.INS1092471.

The holotype is in good condition and is deposited, together with one male and female paratype in the National Biodiversity Center, Bhutan (NBCB). The remaining three paratype males, as well as the DNA material are stored in the collection of Naturalis Biodiversity Center, the Netherlands (NBC).

#### Remarks.

The male of *Eumerus druk* Smit, sp. nov. is easily distinguished from all other species in the *bactrianus* subgroup by the long, silvery, ventrally directed, pile on the lateral sides of t3 and t4. *Eumerus banaticus* has some longer pile on the lateral sides of t4, but this is shorter, not ventrally directed, and not present on t3. Furthermore *E. banaticus* is easily distinguished by the lack of pollinose maculae on t4 and by the shape of st4 and the terminalia. *Eumerus hungaricus* Szilády, 1940 and *E. pulchellus* Loew, 1848, which have similar long, ventrally directed pile on t3 and t4, are superficially similar but the pile is much more dense. *Eumerus druk* Smit sp. nov. can easily be distinguished by the bifurcate posterior surstyle lobe. *E. hungaricus* and *E. pulchellus* furthermore lack the golden-coppery luster on the thorax and abdomen of *E. druk*. *Eumerus pulchellus* is a more slender built species, with a more bluish luster, a relatively slender metafemur, the pro- and mesotarsi predominantly light brown, the basoflagellomere orange. *Eumerus hungaricus* is a more black species with less luster, especially on the abdomen, which is predominantly black pilose; s3 is very slender, about 2.5 times longer than wide, and t4 has a yellow posterior margin, medially.

### Eumerus turanicola

Taxon classificationAnimaliaDipteraSyrphidae

Stackelberg, 1952

B16CCF1C-E566-51D4-AEFD-CFE8A215258A

#### Diagnosis.

(based on Stackelberg 1952). Body golden- or bronze-green. Legs dark bronze-green, tip of the femora, basal half as well as the tips of the tibia and tarsi reddish yellow. Metaleg with basotarsomere not expanded nor shortened, longer than second and third segment combined. Basoflagellomere triangular (Fig. 2H). s4 figured by Stackelberg (1952, 1961), gradually widening posteriorly, with a broad incision posteriomedially, with two sharp angles well below the apex of S4 and two rounded lobes on both sides of the incision. Male terminalia figured by Stackelberg (1952, 1961), posterior surstyle lobe with a ventral triangular extension.

### Eumerus turanicus

Taxon classificationAnimaliaDipteraSyrphidae

Stackelberg, 1952

5FF80C78-DA82-5EF2-8A38-3C01758EEA2C

#### Diagnosis.

(based on Stackelberg 1952). Body bronze-green. Legs dark bronze green, tip of the femora, basal half as well as the tips of the tibia and tarsi reddish yellow. Metaleg with basotarsomere not expanded nor shortened, longer than second and third segment combined. Basoflagellomere oval-shaped (Fig. 2I). s4 figured in Stackelberg (1952, 1961), gradually narrowing, with an incision posteriomedially, with two rounded, densely pilose angles at the apex of s4, with two rounded, spatulate lobes on both sides of the incision. Male terminalia figured in Stackelberg (1952, 1961), with dense pilosity on the main branch of the posterior surstyle lobe, cerci with distinct thorn-like projections.

##### An identification key to the males of the Central and Eastern Palaearctic species of the *Eumerus bactrianus* subgroup

**Table:** 

1	All tarsi entirely black. Basoflagellomere rectangular (Fig. 2G), with a rounded posterior corner. Abdomen with t2 and t3 shiny black in the middle, amplified by the short adpressed black pile, continuing on the basal part of t4. Abdominal t3 and t4 with long, silvery, ventrally directed, white pile on the lateral sides, s4 without an incision posteriomedially, but middle part of sternite less sclerotized. Terminalia: posterior surstyle lobe with a tuft of long pile just anteriorto the bifurcation	***E. druk* Smit, sp. nov.**
–	Tarsi predominantly reddish-yellow. Basoflagellomere trapezoid, oval or triangular shaped. Abdomen with t2–4 with the same bronze-green luster as laterally. Male: s4 with a clear incision posteromedially and posterior surstyle lobe lacking the tuft of long pili prior to the bifurcation	**2**
2	Basoflagellomere trapezoid (Fig. 2F). s4 without lobes on both sides of the posteriomedially incision, with a deep and sharp notch. Terminalia: anterior surstyle lobe elongated, ventral margin of posterior surstyle lobe greatly produced	***E. bactrianus* Stackelberg**
–	Basoflagellomere not trapezoid but oval or triangular. s4 with a deep, angular incision posteriomedially	**3**
3	Basoflagellomere triangular (Fig. 2H). s4 gradually widening posteriorly, with a broad incision posteriomedially, with two sharp angles well below the apex of s4 and two rounded lobes on both sides of the incision. Terminalia: posterior surstyle lobe with a ventral triangular extension	***E. turanicola* Stackelberg**
–	Basoflagellomere oval (Fig. 2I). s4 gradually narrowing posteriorly, with an incision posteriomedially, with two rounded, densely pilose angles at the apex, with two rounded, spatulate lobes on both sides of the incision. Terminalia: with dense pilosity on the main branch of the posterior surstyle lobe, cerci with distinct thorn-like projections	***E. turanicus* Stackelberg**

## Supplementary Material

XML Treatment for Eumerus bactrianus

XML Treatment for Eumerus druk

XML Treatment for Eumerus turanicola

XML Treatment for Eumerus turanicus
